# Evaluation of Heat Stress Levels Inside Greenhouses during Summer in Korea

**DOI:** 10.3390/ijerph191912497

**Published:** 2022-09-30

**Authors:** Wongeon Jung, Hyocher Kim

**Affiliations:** National Institute of Agricultural Sciences, Rural Development Administration, 310 Nongsaengmyeong-ro, Deokjin-gu, Jeonju-si 54875, Korea

**Keywords:** WBGT, farming, greenhouse, heat stress

## Abstract

Farmers working inside greenhouses during summer are at risk of heat-related illness. In this study, we compared the dry-bulb temperatures (DTs) and the wet-bulb globe temperature (WBGT) index inside and outside greenhouses. We then determined the criteria for appropriate working and resting times inside greenhouses. The measurements were performed during the hottest period in 2020 and 2021 for two greenhouses, representative of those commonly found in rural areas. A direct-reading WBGT index meter was used for these measurements, and Automated Synoptic Observing System (ASOS) data were used to obtain regional DT data. DTs inside the greenhouses were significantly higher than the ASOS DTs (*p* < 0.001). In addition, the August WBGT index inside was significantly higher than that outside the greenhouses (*p* < 0.001). We found that the temperature during the period between 08:00 and 19:00 exceeded the international threshold of 28 °C for heat-acclimatized workers performing moderate-intensity work, above which heat stress becomes a concern. Our results revealed that the thermal index inside can be significantly higher than that found outside greenhouses. Therefore, when work is required inside greenhouses during the summer, stricter standards and safety measures should be considered.

## 1. Introduction

Increasing temperatures, caused by global warming and climate change, are significantly affecting the agricultural sector, especially with regards to land use and food supply [1,2]. Extremely high temperatures not only affect crop growth but are also dangerous for vulnerable individuals, such as people with chronic diseases, children, and the elderly [3,4]. Seasonal and average annual temperatures in Korea have also increased gradually [5]. In Korea, 1376 people suffered from heat-related illnesses during the summer of 2021; these included 109 workers engaged in the agriculture, forestry, and fishing sectors, representing approximately 7.9% of these individuals [6].

Agricultural work is mostly performed outdoors or in facilities such as greenhouses, which can be directly affected by the outdoor temperature. Thus, farmers are highly likely to be exposed to heat stress, and elderly farmers can be even more vulnerable. In a survey on occupational diseases and injuries among farmers conducted in Korea in 2018, among all the respondents (1,864,444) who experienced abnormal health symptoms during agricultural work, approximately 6.9% (128,814) experienced headache or dizziness, and among them, approximately 56.2% (72,358) of the farmers responded that indoor and outdoor heat was the cause of their ailment. In addition, 73.2% (94,337) of all respondents reported experiencing such symptoms from July to September. Based on the data from 2016, 71.2% of a total of 112,569 respondents who experienced headache or dizziness experienced those symptoms during work in the fields, and approximately 6.8% (7698 respondents) experienced such symptoms during work in facilities [7].

Because heat stress can have severe health effects, various techniques have been suggested to evaluate heat stress. It is important to examine the level of heat exposure in hot environments because such environments can affect cardiorespiratory, mental, and renal health, and pregnancies, and can also cause heat-related illnesses, manifesting, for example, as stroke, exhaustion, cramps, collapse, and fatigue [3,8,9,10]. Indices available for evaluating heat stress include the wet-bulb globe temperature (WBGT), discomfort index, predicted heat strain, and universal thermal climate index [11,12]. In particular, the WBGT has been utilized in various fields, including sports and clothing, as it is a representative tool to evaluate the heat exposure level of individuals [13]. The WBGT has also been used to evaluate hot work environments in industrial fields.

In the agricultural sector, the heat exposure level of farmers during agricultural work has been evaluated [14,15,16], but only a few studies have evaluated heat stress inside greenhouses. In Korea, protected cultivation is mostly performed in polyethylene-covered greenhouses [17]. As of 2020, greenhouses represented approximately 99.3% (52,055 ha) of Korea’s protected cultivation area (52,444 ha) [18]. In summer, the temperature inside these greenhouses can be similar to or higher than the outdoor temperature, owing to the properties of the material used in the construction or insufficient ventilation. Most farmers depend on thermo-hygrometers installed in greenhouses for the environmental management required for the growth of crops and weather forecasts and check only the dry-bulb temperature level. Recently, the prevalence of work environments such as smart farms, wherein the temperature and humidity are controlled automatically, has increased. However, even in such farms, only the temperatures relevant to crops are measured and controlled, and there are limitations in identifying the level of heat stress for farmers. Although there are some standards related to WBGT [19], it is difficult to determine the work intensity or rest time by applying these standards when an accurate index is unavailable. Therefore, it is necessary to quantify the level of heat stress that farmers can be exposed to in the summer inside typical greenhouses.

Accordingly, this study was aimed at evaluating the heat stress level that farmers could be exposed to during the summer of 2020 and 2021, when working inside typical greenhouses. To this end, the dry-bulb temperature (DT) and WBGT index were measured inside and outside greenhouses and were then compared with the DTs in the Automated Synoptic Observing System (ASOS) data provided by the Korea Meteorological Administration (KMA), which can be accessed by farmers through various media. In addition, the pattern of temperature change was analyzed through time-series data, and the thermal index was examined for each time period in summer to identify the appropriate preventative measures for each work hour when working in typical greenhouses.

## 2. Materials and Methods

### 2.1. Study Design

**Figure 1:**
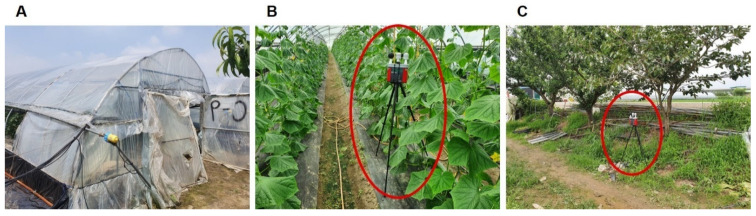
Sampling location. (**A**) Typical greenhouse. (**B**) Wet-bulb globe temperature (WBGT) sampling inside the greenhouse. (**C**) WBGT sampling outside the greenhouse.

This study was conducted in the summer of 2020 and 2021. Measurements were performed from June to September, a period that includes the hottest months, July and August. Two greenhouses (ID: N and W), in which zucchini, cucumbers, cherry tomatoes, and red peppers were mainly grown, were targeted. During the measurement period, all the tasks required to cultivate crops, such as plowing, planting, rowing and fixing, spraying fertilizer, installing shading net, suckering, vinyl removal, spraying pesticide, harvesting, packing, and delivery, were studied. Measurement-related information is presented in Table 1, and Figure 1 shows the target greenhouse.

### 2.2. Data Collection

#### 2.2.1. Sampling Inside and Outside Greenhouses

Both single-span and multi-span greenhouses were included in the study. The dimensions of the multi-span greenhouse (ID: N) were 6 m (W) × 90 m (L) × 2.5 m (H) and its volume was 1350 m^3^. The dimensions of the single-span greenhouse (ID: W) were 17 m (W) × 100 m (L) × 2.5 m (H) and its volume was 4250 m^3^. The greenhouses had steel frames covered with a plastic material, and there was one or no fan installed on the ceiling as a ventilation device. Windows on the sides of the greenhouses were mostly open during the summer, and most of the work was performed between 04:00 a.m. and 11:00 a.m. The sampling inside the greenhouse was performed at a location in the center of the indoor area, and the outdoor sampling location was in a non-shaded area, approximately 5 m away from the entrance of the greenhouse. Two measuring devices were used: one installed indoors and another installed outdoors. The WBGT was measured by installing a monitoring device at a height of approximately 1.5 m from the floor, and it was similarly measured outside the greenhouse (Figure 1). A thermal environmental monitor (model: QUESTemp° 48N, TSI Inc., Shoreview, MN, USA) was used for the WBGT measurements, and the interval time was set to 1 min. The accuracy and range of the device (with dry-bulb or globe sensors) were ± 0.5 and 0–120 °C, respectively. The waterless wet bulb (humidity) sensor had a higher measurement uncertainty, with an accuracy of 1.1 °C (k = 2) in the range between 0 and 80 °C. All the collected data were processed into 1 h average data.

#### 2.2.2. Automated Synoptic Observing System (ASOS) Data

The ASOS data, provided by the KMA, were used for the collection of weather data. The ASOS data are obtained from simultaneous ground observations at all observatories installed across the country to identify atmospheric conditions at specific times of the day. Atmospheric conditions such as the temperature, precipitation, humidity, wind direction, and wind speed are automatically observed. The ASOS data were selected because they could represent atmospheric conditions in the observed area and were the same local forecast data that could be accessed by farmers. Among the ASOS data corresponding to the area containing the target greenhouses at the same measurement time, we referred to the DT data at 1 h intervals.

### 2.3. Data Analysis

The collected data were analyzed using descriptive statistics. An exploratory data analysis was performed and the normal distribution of data was checked using the Shapiro–Wilk and Kolmogorov–Smirnov tests. The data were not normally distributed. However, because the number of samples was 30 or more, it satisfied the central limit theorem (CLT), and we could use the parametric test method (i.e., paired *t*-test) [20]. The paired *t*-tests were used to compare the ASOS data and DTs inside and outside the greenhouses and the WBGT inside and outside the greenhouses by month. We used the SPSS Statistics software (version 27, IBM Corp., New York, NY, USA) for all statistical analyses.

## 3. Results

### 3.1. Comparison of ASOS Data and Dry-Bulb Temperatures Inside and Outside Greenhouses by Month

Table 2 shows the data from the greenhouses and the DT from the ASOS data by month. The highest average DT inside the greenhouses in 2020 was in August (30.3 ± 6.0 °C), and in 2021, it was in July (29.4 ± 5.9 °C). The highest average DT in 2020 from the ASOS data was in August (28.2 ± 2.5 °C), and in 2021, it was in July (28.7 ± 2.9 °C). On comparing the average DT inside the greenhouses in July and August with those from the ASOS data, the former were found to be significantly higher. The average DT inside the greenhouses in July 2020 was 27.1 ± 5.8 °C, which was significantly higher than that from the ASOS data (23.4 ± 2.6 °C) (*p* < 0.001). The average DT inside the greenhouses in August 2020 was 30.3 ± 6.0 °C, which was significantly higher than that from the ASOS data (28.2 ± 2.5 °C) (*p* < 0.001). Similarly, the average DT inside the greenhouses in July 2021 (29.4 ± 5.9 °C) was significantly higher than that from the ASOS data (28.7 ± 2.8 °C) (*p* < 0.05), and the average DT inside the greenhouses in August 2021 (28.0 ± 7.0 °C) was significantly higher than that from the ASOS data (25.8 ± 3.1 °C) (*p* < 0.001). The average DT outside the greenhouses was similar to the ASOS data, and it was generally lower than the average DT inside the greenhouses.

### 3.2. Comparison of WBGT Levels Inside and Outside Greenhouses and the Dry-Bulb Temperature from ASOS Data

#### 3.2.1. WBGT Levels Inside and Outside Greenhouses

Table 3 shows the results of the descriptive statistics of the WBGT index measured inside and outside the greenhouses. The average WBGT index was high in July and August, as in the average DT results presented in Table 2. Overall, the average WBGT index inside the greenhouses was significantly higher than that outside the greenhouses (*p* < 0.001). In 2020, the average WBGT index inside the greenhouses increased to 29.1 ± 4.8 °C in August and then showed a tendency to decrease in September. However, in 2021, the average WBGT index increased to 28.0 ± 4.5 °C in July but decreased to 25.6 ± 4.5 °C in August. The same tendency was observed from the average WBGT index outside the greenhouses. In August 2020 and July 2021, when the average WBGT index inside the greenhouses was the highest, the index reached up to 41.2 and 42.5 °C, resulting in differences of 5.7 and 2.3 °C from the maximum values outside the greenhouses, respectively.

#### 3.2.2. Time-Series Comparison between Greenhouse WBGT and the Dry-Bulb Temperature from ASOS Data

**Figure 2:**
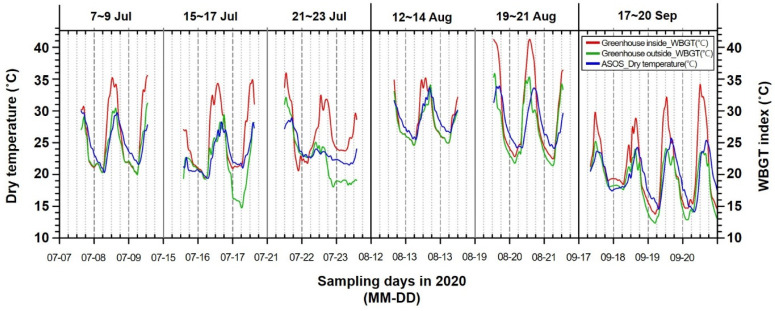
Time-series graphs of the wet-bulb globe temperature (WBGT) index inside and outside greenhouses and of the dry-bulb temperature from Automated Synoptic Observing Systems (ASOS) data for 2020.

**Figure 3:**
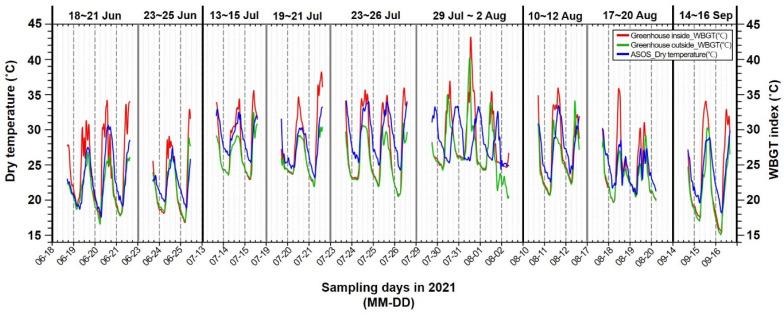
Time-series graphs of the wet-bulb globe temperature (WBGT) index inside and outside greenhouses and of the dry-bulb temperature from Automated Synoptic Observing Systems (ASOS) data for 2021.

Figure 2 and Figure 3 show the average WBGT index inside and outside the greenhouses, as measured in 2020 and 2021, respectively, and the average DT from the ASOS data in time-series graphs. In both the figures, the average WBGT index inside the greenhouses is generally higher than that outside the greenhouses and the DT from the ASOS data. In particular, the average WBGT index inside the greenhouses increased significantly from 08:00 to 18:00, as compared with the average WBGT index outside the greenhouses and the average DT from the ASOS data. The average WBGT index outside the greenhouses was similar to the average DT from the ASOS data.

### 3.3. Greenhouse WBGT Levels by Time Period

**Figure 4:**
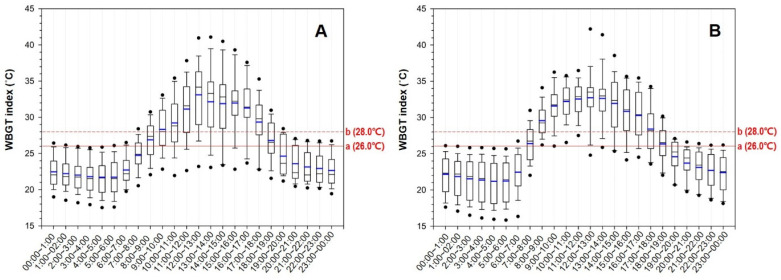
Wet-bulb globe temperature (WBGT) index inside greenhouses over an hourly interval (June–September in 2020 (**A**) and 2021 (**B**)) with reference lines for moderate work, as defined by ISO 7243, including a solid line, (a), showing the reference limit for persons not acclimatized to heat, and a dashed line, (b), showing the reference limit for persons acclimatized to heat.

Figure 4 shows the average indoor WBGT index data from June to September at 1 h intervals for a day. The highest average WBGT index values between 12:00 and 13:00 were 33.1 ± 4.7 and 32.7 ± 3.8 °C for 2020 and 2021, respectively. In addition, the maximum WBGT index during this time period reached 41.2 and 42.5 °C in 2020 and 2021, respectively. The time periods with an average WBGT index of 30 °C or higher were 11:00 to 17:00 in 2020 and 09:00 to 17:00 in 2021. For domestic agricultural work characterized as moderate work, the reference limits provided by the heat stress standard ISO 7243 for workers acclimatized to heat and those not acclimatized to it were considered. When 28 °C was considered as the reference limit of heat-acclimatized workers, this limit was exceeded from 09:00 to 18:00 in 2020 and from 08:00 to 18:00 in 2021. For the assumption that the domestic farmers are heat-acclimatized workers, we used a reference limit of 28 °C, and found that these workers were commonly exposed to heat stress from 08:00 to 19:00.

## 4. Discussion

Heat stress can be increased by various environmental variables, including temperature, and can cause various heat-related illnesses. Farmers mostly work outdoors or in greenhouses and are at risk of experiencing heat stress. In greenhouses, the heat stress may be significant because solar heat is trapped inside, and the application of heat reduction measures that do not interfere with crop growth is difficult. In this study, the indoor and outdoor WBGT index levels from June to September—the hottest months—in 2020 and 2021 were evaluated for greenhouses in Korea; they were compared with DT data from the national ASOS data. In general, our findings show that the heat-stress-inducing temperatures experienced by greenhouse workers were not exactly proportional to the outdoor temperatures. The DTs inside the greenhouses were significantly higher than those from the ASOS data and the DTs outside the greenhouses, and the interior WBGT index was also higher than that on the exterior. In particular, the WBGT index was the highest in July and August, which are the two hottest months, and the average WBGT index increased to more than 30 °C and exceeded 40 °C from 12:00 to 17:00.

The results obtained in this study were affected by several variables, including the weather, greenhouse characteristics, and the presence or absence of a ventilation device. The weather was generally clear during the measurement period. The side windows and the windows in the ceiling were open at all times in 2020; they opened automatically at 25 °C in the same manner during the measurement period in 2021. Overall, the WBGT index inside the greenhouses increased most significantly from 08:00 to 18:00 and was significantly different from that outside the greenhouses. Zucchini and cucumbers were the main crops grown in the target greenhouses; however, the results that showed the smallest difference between the inside and outside were measured from 12–14 August 2020 and were obtained from the cherry tomato greenhouse. Although the crop height of the zucchini and cucumbers was approximately 2 m, the cherry tomatoes reached the ceiling at the time of the measurements in the greenhouse. The shade created by these tall crops may have reduced the measured temperature, thereby affecting the results. It is, therefore, possible that a more significant difference in the WBGT could have been measured in the absence of this effect.

A comparison with previous studies, in which the heat exposure level of workers was evaluated, shows that workers can be exposed to high heat stress inside greenhouses. The heat exposure level in outdoor work, such as in construction, increases to an average of 33 °C or a maximum of more than 40 °C, approximately between 10:00 and 12:00, when the sunlight is the strongest [21]. In this study, the temperature also increased to an average of more than 30 °C from 08:00 to 11:00, and to an average of approximately 33 °C and a maximum of 41.2 °C between 12:00 and 13:00 before decreasing. Because thermal energy is conserved inside the greenhouses, it was highly likely to remain at an average of more than 30 °C until approximately 17:00, unlike in the case of outdoor work. The temperature can also exceed 30 °C during specific time periods, even in June and September, as indicated by our results, even though July and August are expected to be the hottest, warranting the exercise of caution. Farmers who work outdoors in the period from April to October can be exposed to a maximum of 33–38 °C [16], but a direct comparison is difficult because regional and weather conditions are different.

To prevent heat-related illnesses, studies were conducted on the relationship between the metabolic equivalent of task (MET) and the WBGT index, and exposure limits were set and presented nationally and internationally. The International Organization for Standardization presents appropriate WBGT index criteria by job classification, according to the MET, for both heat-acclimatized and unacclimatized persons [19]. The work performed inside greenhouses was investigated, and most of these jobs were assumed to correspond to moderate work (class 2). Compared with 28 °C, a criterion for heat-acclimatized persons, the temperature for each time period was higher from 08:00 to 18:00 in both 2020 and 2021, that is, for 10 h a day.

**Figure 5:**
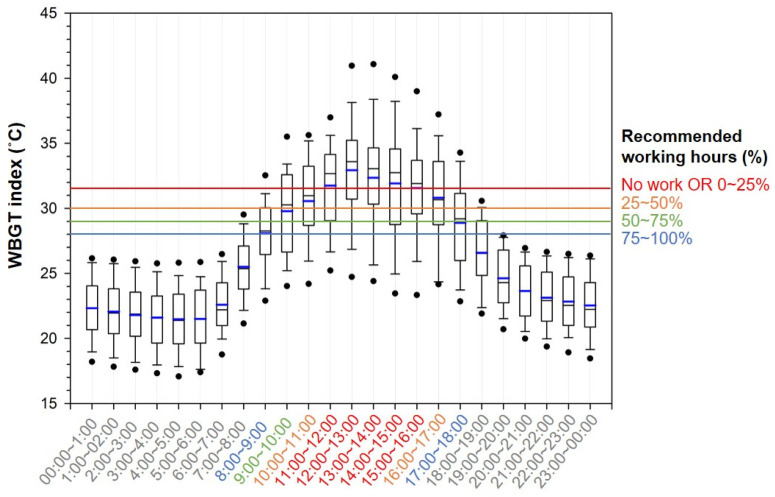
Recommended working hours during daily time in the greenhouse according to threshold limit values for moderate work (ACGIH, 2019).

Figure 5 shows the results of applying the threshold limit value (TLV) of the WBGT index, provided by the American Conference of Governmental Industrial Hygienists (ACGIH), to the results obtained in this study. The ACGIH sets the WBGT index for each of the four work intensities (light, moderate, heavy, and very heavy) according to the MET and suggests an appropriate ratio between working and rest hours [22]. In the results obtained in this study, the moderate criterion of TLV was applied, considering the work characteristics inside domestic greenhouses. The ratio of working hours was found to be 0% to 25% for 11:00 to 16:00, 25% to 50% for 10:00 to 11:00 and 16:00 to 17:00, 50% to 75% for 09:00 to 10:00, and 75% to 100% for 08:00 to 09:00 and 17:00 to 18:00. In other words, per hour of work performed during the time period from 11:00 to 16:00, the maximum working time must be limited to 15 min with 45 min of rest. When the results of this study are considered conservatively (based on the maximum values rather than the average values), it is likely that the temperature exceeds 31.5 °C and workers are exposed to heat stress from 08:00 to 18:00. In particular, a maximum of 40 °C can be exceeded from 12:00 to 15:00, and it is judged appropriate to interrupt work during this time period because the risk may vary depending on the level of heat acclimatization, clothing, work intensity, and sensitivity of an individual worker. Therefore, it is recommended to plan work in the early morning or late afternoon. If work during the more dangerous time period is considered, the rest time must be set to 75% of the total working hours or more.

Appropriate working and rest hours were considered by applying the results of this study to several criteria, but the WBGT index criteria may differ depending on individual characteristics. Heat-related illness is related to various risk factors, such as obesity, dehydration, weak physical condition, a history of heat-related illness, lack of acclimatization, and hydration level [8]. Moreover, old farmers can be more vulnerable to heat stress, and the risk of heat-related illness can be higher for people aged over 60 years and suffering from chronic diseases [23]. As of 2021, the number of farmers aged over 60 years in Korea was estimated to be 1,381,854, which accounts for approximately 62.4% of all farmers in Korea [24]. Therefore, it is necessary to conduct research and implement measures to reduce heat stress to protect these farmers, particularly those working in greenhouses.

Greenhouses offer protected cultivation and carefully maintained conditions to promote crop growth. The air temperature and relative humidity inside greenhouses can be greatly affected by the type of ventilation window and external wind speed. The temperature inside a greenhouse may vary depending on several variables [25]. When ventilation fans are installed, the air temperature may change at windward or leeward points [26]. However, in such studies on environmental conditions in greenhouses, the focus is on the growth of crops and not on the exposure of farmers to heat stress. Based on previous studies, it is judged that there are limitations in seeking ways to reduce the WBGT of farmers by reducing the air temperature through the ventilation of greenhouses. Therefore, when farmers work in greenhouses in summer, it is considered effective to devise a strategy to provide proper rest hours during specific time periods and a method that allows farmers to reduce heat themselves. In addition, plant factories, which have been expanded recently, can be helpful in managing workers’ heat stress. Ambient environment factors inside plant factories, such as light, temperature, humidity, air velocity, and carbon dioxide, can be set for the growth of specific crops [27]. In addition, because the work intensity inside plant factories is judged to be relatively lower than that found inside greenhouses [28], a reduction in the MET is expected to have a positive impact on the WBGT index. If plant factories continue to be used in the future, it will be necessary to consider the safety and health of farmers by combining a system that evaluates the thermal index of workers with the monitoring of ambient environmental factors.

Farmers in Korea use media, such as weather forecasts or simple thermo-hygrometers, to monitor the environment inside greenhouses. This has limitations in evaluating the thermal index that may vary depending on several variables. In addition, there are insufficient institutions or experts to evaluate the thermal index in the agricultural fields of Korea, and relevant education is not sufficient for farmers. Because the elderly farmers represent more than 50% of all farmers in Korea, it can be difficult to provide education and communication regarding the measurement and evaluation of the thermal index, which should be used to determine working hours by taking into consideration the relationship between labor intensity and the WBGT index. Therefore, the results of this study will be used as basic data to inform farmers about the time periods to be avoided for work in greenhouses in summer and the proper setting of the recommended ratio between working and resting time for each time period.

The types of protected cultivation include plastic or glass greenhouses, shade net houses, and plant factories; the type of protected cultivation used differs among countries. Several European countries (e.g., Northern Europe) mainly use glass greenhouses and the others use plastic-film-covered greenhouses (e.g., South of France) [29]. Although this study was targeted at plastic greenhouses (i.e., polyethylene-covered greenhouses) mainly used in Korea, it is deemed necessary to evaluate the heat stress in the other types, considering the increase in temperature is expected to continue because of recent climate changes [30]. This should be taken seriously because an increase in the risk of heat illness among farmers is expected. The results of this study might be useful in predicting heat stress by monitoring the WBGT index. Research on the exposure of farmers to heat stress should be continued for various protected cultivation types using different methods for the evaluation. Hence, this study marks the beginning of the research on the heat stress exposure of farmers in greenhouses. We believe that our study is the preliminary research on the exposure of farmers to heat stress in greenhouses. The methodology employed by us can be used as a reference for other studies, for educating farmers, or for establishing policy standards. Moreover, heat stress is a serious problem in low-latitude developing and underdeveloped countries. The present study will be helpful to researchers in countries other than Europe and North America who are interested in conducting such investigations.

In this study, two greenhouses in Korea were evaluated. Therefore, there are limitations in extrapolating these data to greenhouses in regions or places with different climatic conditions. Although studies were conducted on the temperature, wind direction, wind speed, humidity, and thermal energy in greenhouses, a study on the heat stress experienced by workers was required. The results of this study confirm that the heat stress level inside greenhouses is high. Because the WBGT index evaluates heat stress through the temperature, relative humidity, radiant heat, and airflow, a quantitative assessment that includes all these variables inside greenhouses must be performed in the future. In addition, it is necessary to evaluate and compare the heat stress levels under various farm settings, such as in paddies, fields, and orchards. In this study, the criteria for each time period in the warm season were proposed by collecting data from June to September for the number of samples. Therefore, securing enough samples in the future will be helpful in preparing criteria for determining daily working hours depending on the time period by analyzing monthly measurements.

## 5. Conclusions

In this study, the heat stress level inside greenhouses in summer in Korea was evaluated. The dry-bulb temperature and wet-bulb globe temperature (WBGT) index were, as expected, the highest in July and August, showing a significant difference from the dry-bulb temperature of weather forecasts. The overall results confirm that the dry-bulb temperature inside greenhouses can be higher than the atmospheric dry-bulb temperature, even in June and September. The WBGT index inside greenhouses was significantly higher than it was outside greenhouses, and a significant difference was observed from 08:00 to 18:00. In the case of modern greenhouses, employing cutting-edge technology wherein the temperature and humidity can be controlled, the indoor WBGT index was found to be relatively lower than the outside or atmospheric data. If the ISO international standards are applied through the results averaged at 1 h intervals, workers unacclimatized to heat are likely to be exposed to heat stress from 08:00 to 18:00. In addition, if the threshold limit value (TLV) of the American Conference of Governmental Industrial Hygienists (ACGIH) is applied under the assumption of moderate work, work must be interrupted or rest time must be provided at a ratio of up to 75% from 12:00 to 16:00. Because the WBGT index or the risk of heat-related illness may vary depending on several variables, appropriate working hours must be set considering the environment and work being performed. In addition, it is necessary to establish proper safety measures for each time period, such as allowing sufficient rest time and working in groups of two, so that workers can monitor each other for signs of heat-related illnesses.

## Figures and Tables

**Table 1:** Sampling information.

Sampling Date			Sampling Time (h)	Product	Task	Region ^(a)^
Year	Month	Day				
2020	6	10–12	50	Zucchini	Harvesting, Packing	N
		16–18	52	Cucumber	Harvesting	W
		22–24	47	Zucchini	Plowing	N
		26–29	72	Cucumber	Rowing	W
	7	1–3	48	Zucchini	Vinyl removal	N
		7–9	47	Cucumber	Rowing	N
		10–13	71	Zucchini	Spraying fertilizer	N
		15–17	50	Cucumber	Harvesting	W
		21–23	51	Zucchini	Installing shading net	N
	8	4–6	45	Red pepper	Vinyl removal	N
		12–14	45	Cherry tomato	Harvesting	W
		19–21	49	Red pepper	Suckering	N
	9	17–21	93	Red pepper	Spraying pesticide	N
2021	6	18–21	71	Cucumber	Planting	W
		23–25	43	Zucchini	Harvesting	W
	7	13–15	47	Zucchini	Harvesting	W
		19–21	47	Cucumber	Harvesting	W
		23–26	70	Zucchini	Stem removal	W
		29–8/2	93	Cucumber	Harvesting	W
	8	10–12	47	Zucchini	Greenhouse maintenance	W
		17–20	58	Cucumber	Harvesting	W
	9	14–16	47	Cucumber	Harvesting	W

^(a)^ W, Wanju-Gun Jeollabuk-do; N, Naju-si Jeollanam-do.

**Table 2:** Comparison of dry-bulb temperatures between greenhouse (inside and outside) measurements and ASOS data.

Year	Month	Greenhouse						ASOS ^(c)^						*p* ^(b)^
		Inside			Outside									
		Dry-Bulb Temperature (°C)			Dry-Bulb Temperature (°C)			Dry-Bulb Temperature (°C)			Relative Humidity (%)			
		*n* ^(a)^	AM ± SD	Min–Max	*n* ^(a)^	AM ± SD	Min-Max	*n*	AM ± SD	Min-Max	*n*	AM ± SD	Min-Max	
2020	06	221	26.4 ± 6.0	17.6–46.2	NA	NA	NA	221	24.0 ± 3.1	18.7–31.5	221	77.2 ± 16.4	36.0–99.0	<0.001
	07	267	27.1 ± 5.8	17.6–43.3	219	24.0 ± 3.2	19.2–32.8	267	23.4 ± 2.6	19.0–30.0	267	86.7 ± 12.4	51.0–99.0	<0.001
	08	139	30.3 ± 6.0	22.5–45.7	94	28.2 ± 4.1	21.5–35.3	139	28.2 ± 2.5	24.1–33.9	139	83.0 ± 12.9	51.0–99.0	<0.001
	09	93	21.2 ± 5.8	12.8–34.1	93	19.4 ± 5.1	11.0–28.2	93	19.6 ± 3.2	14.1–25.7	93	84.1 ± 17.6	41.0–99.0	<0.001
2021	06	114	24.4 ± 5.7	16.9–38.1	114	23.2 ± 4.7	17.0–33.1	114	22.8 ± 3.2	17.6–30.6	114	72.5 ± 15.7	33.0–94.0	<0.001
	07	251	29.4 ± 5.9	20.8–43.6	251	28.1 ± 4.2	20.7–38.2	251	28.7 ± 2.8	23.3–34.0	251	69.3 ± 13.5	42.0–96.0	<0.05
	08	108	28.0 ± 7.0	19.9–45.7	108	25.7 ± 4.6	20.2–37.0	108	25.8 ± 3.1	20.8–33.4	108	71.9 ± 12.1	38.0–92.0	<0.001
	09	48	25.1 ± 7.6	15.6–37.8	48	22.8 ± 5.2	15.4–31.5	48	23.7 ± 3.3	18.3–29.9	48	59.2 ± 9.2	44.0–73.0	<0.05

***Abbreviations*:** AM, arithmetic mean; SD, standard deviation; NA, not available; Min, minimum; Max, maximum. ^(a)^ One sample was averaged every 1 h with 1 min intervals (e.g., “*n* = 221” indicates averaged samples for a measurement time of 221 h). ^(b)^ Paired *t*-test to analyze the statistical significance of the dry-bulb temperature inside the greenhouse and from ASOS data by month. ^(c)^ Automated Synoptic Observing Systems data provided by the Korea Meteorological Administration Open MET Data Portal.

**Table 3:** WBGT indices inside and outside greenhouses.

Year	Month	WBGT (°C) in Greenhouse			WBGT (°C) Outside Greenhouse			*p* ^(b)^
		*n* ^(a)^	AM ± SD	Min–Max	*n* ^(a)^	AM ± SD	Min–Max	
2020	6	221	25.3 ± 5.1	17.6–39.1	NA	NA	NA	NA
	7	267	25.5 ± 4.7	17.3–36.0	219	22.7 ± 3.7	14.8–32.1	<0.001
	8	139	29.1 ± 4.8	22.4–41.2	94	27.5 ± 3.7	21.4–35.5	<0.001
	9	93	20.9 ± 5.6	12.8–34.2	93	18.4 ± 3.9	10.9–25.0	<0.001
2021	6	114	23.7 ± 4.9	16.8–34.0	114	21.6 ± 2.9	16.9–28.3	<0.001
	7	251	28.0 ± 4.5	20.7–42.5	251	26.4 ± 3.3	20.3–40.2	<0.001
	8	108	25.6 ± 4.5	19.7–35.8	108	24.2 ± 3.1	19.8–34.0	<0.001
	9	48	23.5 ± 6.2	15.6–34.1	48	21.1 ± 4.3	15.1–30.1	<0.001

***Abbreviations*:** AM, arithmetic mean; SD, standard deviation; NA, not available; Min, minimum; Max, maximum; WBGT, wet-bulb globe temperature. ^(a^^)^ One sample was averaged every 1 h with 1 min intervals (e.g., “*n* = 221” indicates averaged samples for a measurement time of 221 h). ^(b)^ Paired *t*-test to analyze the statistical significance of the WBGT index inside and outside the greenhouse by month.

## Data Availability

Not applicable.

## References

[1] Howden S.M., Soussana J.-F., Tubiello F.N., Chhetri N., Dunlop M., Meinke H. (2007). Adapting agriculture to climate change. Proc. Natl. Acad. Sci. USA.

[2] Mahato A. (2014). Climate change and its impact on agriculture. Int. J. Sci. Res. Publ..

[3] Kovats R.S., Hajat S. (2008). Heat stress and public health: A critical review. Annu. Rev. Public Health.

[4] Vicedo-Cabrera A.M., Scovronick N., Sera F., D Royé R.S., Tobias A., Astrom C., Guo Y., Honda Y., Hondula D.M., Abrutzky R. (2021). The burden of heat-related mortali-ty attributable to recent human-induced climate change. Nat. Clim. Chang..

[5] Statistics Korea. http://www.index.go.kr/potal/main/EachDtlPageDetail.do?idx_cd=1400.

[6] Korea Disease Control and Prevention Agency (KDCA) 2021 Annual Report on the Notified Patients with Heat-Related Illness in Korea. https://kdca.go.kr/filepath/boardDownload.es?bid=ATT&list_no=717498&seq=2.

[7] Korean Statistical Information Service (KOSIS). https://kosis.kr/index/index.do.

[8] Gubernot D.M., Anderson G.B., Hunting K.L. (2014). The Epidemiology of Occupational Heat-Related Morbidity and Mortality in the United States: A Review of the Literature and Assessment of Research Needs in a Changing Climate. Int. J. Biometeorol..

[9] Zamanian Z., Sedaghat Z., Hemehrezaee M., Khajehnasiri F. (2017). Evaluation of environmental heat stress on physiological parameters. J. Environ. Heal. Sci. Eng..

[10] Ebi K.L., Capon A., Berry P., Broderick C., de Dear R., Havenith G., Honda Y., Kovats R.S., Ma W., Malik A. (2021). Hot weather and heat extremes: Health risks. Lancet.

[11] U.S. National Institute for Occupational Safety and Health (NIOSH) (2016). Criteria for A Recommended Standard: Occupational Exposure to Heat and Hot Environments.

[12] Gao C., Kuklane K., Östergren P., Kjellstrom T. (2018). Occupational heat stress assessment and protective strategies in the context of climate change. Int. J. Biometeorol..

[13] Budd G.M. (2008). Wet-bulb globe temperature (WBGT)—Its history and its limitations. J. Sci. Med. Sport.

[14] Crowe J., Wessseling C., Solano B.R., Umaña M.P., Ramírez A.R., Kjellstrom T., Morales D., Nilsson M. (2013). Heat exposure in sugarcane harvesters in Costa Rica. Am. J. Ind. Med..

[15] Marucci A., Monarca D., Cecchini M., Colantoni A., Giacinto S.D., Cappuccini A. (2013). The heat stress for workers employed in a dairy farm. J. Agric. Eng..

[16] Frimpong K., Van Etten E.J., Oosthuzien J., Nunfam V.F. (2017). MPHIL Development Studies. Heat exposure on farmers in northeast Ghana. Int. J. Biometeorol..

[17] Hong S.-W., Lee I.-B., Hwang H.-S., Seo I.-H., Bitog J.P., Yoo J.-I., Kim K.-S., Lee S.-H., Yoon N.-K., Kim K.-W. (2008). Numerical Simulation of Ventilation Efficiencies of Naturally Ventilated Multi-Span Greenhouses in Korea. Trans. ASABE.

[18] Korean Ministry of Agriculture, Food and Rural Affairs (MAFRA) (2020). Vegetable Greenhouse and Production Status in 2020 (11-1543000-000051-10).

[19] (2017). Ergonomics of the Thermal Environment—Assessment of Heat Stress Using the WBGT (Wet Bulb Globe Temperature) Index Third Edition.

[20] Julious S.A. (2010). Sample Sizes for Clinical Trials.

[21] Al-Bouwarthan M., Quinn M.M., Kriebel D., Wegman D.H. (2019). Assessment of heat stress exposure among construction workers in the hot desert climate of Saudi Arabia. Ann. Work. Expo. Health.

[22] American Conference of Governmental Industrial Hygienists (ACGIH) (2019). 2019 Threshold Limit Values for Chemical Substances and Physical Agents and Biological Exposure Indices.

[23] Kenny G.P., Yardley J., Brown C., Sigal R.J., Jay O. (2009). Heat stress in older individuals and patients with common chronic diseases. Can. Med Assoc. J..

[24] Korean Statistical Information Service (KOSIS). https://kosis.kr/statHtml/statHtml.do?orgId=101&tblId=DT_1EA1011&vw_cd=MT_ZTITLE&list_id=F_5_1_1&scrId=&seqNo=&lang_mode=ko&obj_var_id=&itm_id=&conn_path=MT_ZTITLE&path=%252FstatisticsList%252FstatisticsListIndex.do.

[25] Ould Khaoua S.A., Bournet P.-E., Migeon C., Boulard T., Chassériaux G. (2006). Analysis of Greenhouse Ventilation Efficiency based on Computational Fluid Dynamics. Biosyst. Eng..

[26] Teitel M., Ziskind G., Liran O., Dubovsky V., Letan R. (2008). Effect of wind direction on greenhouse ventilation rate, airflow patterns and temperature distributions. Biosyst. Eng..

[27] Islam S., Reza M.N., Chowdhury M., Chung S.O., Choi I.S. (2021). A review on effect of ambient environment factors and monitoring technology for plant factory. Precis. Agric. Sci. Technol..

[28] Schmitz A., Moss C.B. (2015). Mechanized agriculture: Machine adoption, farm size, and labor displacement. AgBioForum.

[29] von Elsner B., Briassoulis D., Waaijenberg D., Mistriotis A., von Zabeltitz C., Gratraud J., Russo G., Suay-Cortes R. (2000). Review of Structural and Functional Characteristics of Greenhouses in European Union Countries: Part I, Design Requirements. J. Agric. Eng. Res..

[30] Intergovernmental Panel on Climate Change (IPCC) (2022). Technical summary in Climate change 2022: Impact, Adaptation and Vulnerability (IPCC Sixth Assessment Report), Contribution of Working Group II to the Sixth Assessment Report of the Intergovernmental Panel on Climate Change.

